# Obituary

**Published:** 1881-08

**Authors:** 


### OBITUARY.

Died in Toledo, Thursday, June 30th, Dr. Joseph L. Burger, D. D. S., at the residence of his brother. Dr. B. was 26 years of age, a graduate of the Dental College of the University of Michigan. He was a man of more than ordinary ability and promise. He was a victim of consumption, from which he had been suffering for ten months. He endured his suffering with fortitude and resignation. He was a native of Bavaria.

